# Items

**Published:** 1876-03

**Authors:** 


We have received the following communication, to which we would call the attention of medical students residing in the city and near vicinity, advising them to take advantage of the offer so kindly extended to them:

DEAR DOCTOR:

Be so kind as to state in the JOURNAL that I shall have Surgical Clinics every Saturday, at 11 o'clock A. M., at the General Hospital, during my term of service, to which all students of Medicine are invited.

Very truly yours, CHAS. C. F. GAY.

DISCOURAGING. To the mass of medical graduates, the following from the introductory address of Mr. Davy, at the Westminister Hospital, will not be a very encouraging prospect: "In conclusion, let me firmly assure you that your early prospects in life are most disheartening; your toil and ambition may be equally great, but your pay and dignity will be equally small; you have to deal with an ignorant and proud people, and I advise every one to resign at once any and every thought of becoming a medical man unless he possesses three qualifications: 1. Independence; 2. An aptitude for and a love of the profession; 3. The readiness to pay a heavy premium in this world for his reward in the next. ——ROMANCE IN MEDICINE. Prof. Billroth, in his recent book on Medical Education, which has attracted so much atttention, indulges in some severe strictures upon the Jewish students in Vienna. The point which he urges the most forcibly, and, in fact, the strongest one, is their lack of early education; but a point which he makes, that they are unfit for the study of medicine because devoid of *romance*, will cause a smile among practical, every-day American physicians. ——SCIENCE AND POETRY. The Philadelphia *Ledger*, which, by the way, is unequalled for its poetical obituaries, indulges in the following explanation of a case of Perityphilitis and its result:

A cherry, incompletely ripe,
His little business did for him.
And now, serenely free from gripe,
He is a bob-tailed cherubim.

——LOOKING DOWN ON HIS NEIGHBORS. Prof. Bartholow, of the Medical College of Ohio, in which Cincinnati is situated, or rather, which is situated in Cincinnati, after indulging in considerable glorification over the sucess of the Ohio Medical College, the six thousand patients who are treated annually at the Dispensary for the benefit of the students, but to the sorrow of the younger physicians whom the majority of these six though could and would pay were they not treated free at the Dispensay; the large number students in attendance, etc., etc., calls Dr. Wood, of the Philadelphia *Medical Times*, the terrible name of medical iconoclast, and winds up by deploring the fact that there are so many medical schools, being particularly severe on those in the *villages* of Louisville, Columbus, Cleveland, Indianapolis and Evansville. Dr. Wood must feel severely the epithet of "iconoclast," but who can imagine the feelings of the inhabitants of the above-named places when they learn that what they fondly imagined to be cities of a respectable size are mere villages?——OMISSION. In our list of the prizes awarded at the last Commencement of the Buffalo Medical College, we accidentally omitted to state that a prize of $50 was awarded to E. A. Adams, A. B., by Prof. Mason, for the best examination in Physiology; and one of $25, to C. R. Dryer, for the next best examination.——MISTAKEN IDENTITY. Some time since an article appeared in the *Lancet*, censuring the French Medical press for their blunders in the use of English proper names, citing as an instance an obituary of Huebbenet, which the *Lancet* inferred was meant for Hughes Bennett. The *Gazette Hebdomadaire* replies to the *Lancet*, and points out the fact which that journal had failed to notice, that Huebbenet was a Russian, and that the obituary was in no way applicable to Hughes Bennett.——DR. ANSTIE. The *Practitioner* for January contains a sketch of the life, together with a portrait, of the late Dr. Austie. Dr. T. Lander Brunton succeeds Dr. Austie as Editor of the *Practitioner*.

